# *SETD5* haploinsufficiency affects mitochondrial compartment in neural cells

**DOI:** 10.1186/s13229-023-00550-9

**Published:** 2023-06-01

**Authors:** Mattia Zaghi, Fabiana Longo, Luca Massimino, Alicia Rubio, Simone Bido, Pietro Giuseppe Mazzara, Edoardo Bellini, Federica Banfi, Paola Podini, Francesca Maltecca, Alessio Zippo, Vania Broccoli, Alessandro Sessa

**Affiliations:** 1 Stem Cell and Neurogenesis Unit, Division of Neuroscience, San Raffaele Scientific Institute, Via Olgettina 58, 20132 Milan, Italy; 2 Mitochondrial Dysfunctions in Neurodegeneration Unit, Division of Neuroscience, San Raffaele Scientific Institute, 20132 Milan, Italy; 3 Experimental Neuropathology Unit, INSPE, San Raffaele Scientific Institute, 20132 Milan, Italy; 4 Chromatin Biology and Epigenetics Lab, Department of Cellular, Computational, and Integrative Biology (CIBIO), University of Trento, 38123 Trento, Italy; 5 CNR Institute of Neuroscience, 20129 Milan, Italy; 6 Present Address: University California, Irvine, USA; 7 Present Address: Department of Genetics and Development, Columbia University, New York, NY 10032 USA

## Abstract

**Background:**

Neurodevelopmental disorders (NDDs) are heterogeneous conditions due to alterations of a variety of molecular mechanisms and cell dysfunctions. *SETD5* haploinsufficiency leads to NDDs due to chromatin defects. Epigenetic basis of NDDs has been reported in an increasing number of cases while mitochondrial dysfunctions are more common within NDD patients than in the general population.

**Methods:**

We investigated in vitro neural stem cells as well as the brain of the *Setd5* haploinsufficiency mouse model interrogating its transcriptome, analyzing mitochondrial structure, biochemical composition, and dynamics, as well as mitochondrial functionality.

**Results:**

Mitochondrial impairment is facilitated by transcriptional aberrations originated by the decrease of the SETD5 enzyme. Low levels of SETD5 resulted in fragmented mitochondria, reduced mitochondrial membrane potential, and ATP production both in neural precursors and neurons. Mitochondria were also mislocalized in mutant neurons, with reduced organelles within neurites and synapses.

**Limitations:**

We found several defects in the mitochondrial compartment; however, we can only speculate about their position in the hierarchy of the pathological mechanisms at the basis of the disease.

**Conclusions:**

Our study explores the interplay between chromatin regulation and mitochondria functions as a possible important aspect of *SETD5*-associated NDD pathophysiology. Our data, if confirmed in patient context, suggest that the mitochondrial activity and dynamics may represent new therapeutic targets for disorders associated with the loss of SETD5.

**Supplementary Information:**

The online version contains supplementary material available at 10.1186/s13229-023-00550-9.

## Background

A functional mitochondrial compartment is mandatory to support proper cell physiology and metabolism. This is particularly true for highly metabolic-demanding cells such as neurons. Accordingly, impairments in mitochondrial biogenesis, turnover, and specific functions of the organelle, such as oxidative phosphorylation, calcium handling, and ATP production, are associated with several neurological disorders including Parkinson’s disease, ataxia, epilepsy, and others [1, 2]. Interestingly, mitochondrial dysfunctions have been observed in a slice of cases of neurodevelopmental disorders (NDD) [3–6], for example, the prevalence of mitochondrial disease in children affected by autism spectrum disorders (ASD) was at least 5%, much higher than in general population (0.01%) [7, 8]. However, the etiology and the actual impact of mitochondrial dysfunction in NDD are unclear and poorly investigated. NDDs represent a galaxy of heterogeneous diseases in which genetic causes, when either present or identified, rely mainly on genes encoding for synaptic components, epigenetic modifiers, and transcriptional regulators [9–11]. De novo and familiar mutations in the *SETD5* gene, eventually resulting in genetic haploinsufficiency, have been associated with NDD, specifically ASD and intellectual disability (ID) [10, 12–21]

*SETD5* codifies for a protein containing a SET (Su(var)3–9, Enhancer-of-zeste, Trithorax) domain, a member of the histone-modifying protein family [22]. The direct function of SETD5 as histone methyltransferase activity is debated [23–25]. SETD5 may interact with HDAC-containing complexes regulating access to chromatin [23, 26–28]. Experimental *Setd5* haploinsufficiency in neural stem cells (NSC) and mouse brain affects RNA polymerase elongation rate with consequent alterations on gene transcription at multiple levels including expression level and alternative splicing [29]. Phenotypically, low levels of the protein impact brain development dynamics, synaptic transmission, and potentiation as well as animal behavior possibly resembling what was observed in humans [23, 24, 26]

In this study, we investigated the affinity of SETD5 for mitochondrial-associated genes and identified impairment of mitochondrial compartment in different experimental models of *Setd5* gene loss including NSC and cerebral cortex. We report that a defect in the control of gene transcription leads to alterations in the maturation, dynamics, and functionality of mitochondria that possibly contribute to the etiology of *SETD5*-associated NDDs.

## Methods

### Neural stem cells culture

Neural stem cells (NSCs) were derived from Telencephalic cortex of embryos at 14.5 days of gestation. Embryonic cortices were dissociated, fragmented in Hank’s Balanced Salt Solution (HBSS, Life Technologies) with 1% Penicillin/Streptomycin (Sigma-Aldrich) and digested with papain (10 U/ml, Worthington Biochemical) and cysteine (1 mM, Sigma-Aldrich) in HBSS with 0.5 mM EDTA at 37 °C. The obtained NSCs were routinely cultured in suspension as neurospheres.

Cells were cultured in Neural inducing medium (NIM) composed of: DMEM/F12 (Sigma-Aldrich) supplemented with Hormon Mix (DMEM/F12, 0.6% Glucose (Sigma-Aldrich) (30%), Insulin (Sigma-Aldrich) 250 µg/ml, putrescin powder (Sigma-Aldrich) 97 µg/ml, apotransferrin powder (Sigma Aldrich), sodium selenite 0.3 µM, progesterone 0.2 µM), 1 mg/ml penicillin/streptomycin (Sigma-Aldrich), 2 mM glutamine (Sigma-Aldrich), 0.66% Glucose (30% in phosphate buffer salt (PBS) (Euroclone)), Heparin 4 µg/ml, 10 ng/ml bFGF (basic fibroblast growth factor) (ThermoFisher Scientific) and 10 ng/ml EGF (epithelial growth factor) (10 ng/ml) (ThermoFisher Scientific).

To culture NSCs in adhesion, cells were seeded in coverslips coated with 1:100 Matrigel.

For low oxygen culture condition, cells were maintained in an incubator with 5% oxygen concentration. For high oxygen culture, cells were cultured in an incubator with 21% oxygen concentration.

Mutant *Setd5*+*/*− NSCs were generated as previously explained using Crispr-Cas9 technology [24].

### Glucose-free NSCs culture

NSCs were cultured in normal NIM using DMEM without glucose (GIBCO) instead of normal DMEM. The media was supplemented with 25 mM galactose, Hormon Mix which contains glucose was replaced by N2 supplement, the rest of the ingredients remained the same of NIM.

### RNA extraction with RNAeasy mini kit and RNA sequencing of High oxygen NSCs

To perform RNA extraction, cells were seeded in adherent condition (see above) in 15 mm Petrie dish at approximately 60–70% of confluence and were grown until they reached almost 90% of confluence. Cells were then scraped and pelleted in cold (4 °C) and sterile PBS. RNA extraction was performed using RNAeasy mini kit (Qiagen).

Each sample was lysed using 600 µl of RLT buffer of RLT buffer and 1 volume of 70% ethanol was added and to the cell lysate. Then up to 700 µl of the sample, were transferred to an RNAeasy spin column placed in 2-ml collection tube and centrifuged for 15 s at 10,000 rpm and the flow trough was discarded. If the volume of the sample exceeded 700 µl the centrifuge is repeated a second time with the rest of the sample.

After this step, DNAse digestion is performed to eliminate DNA contaminants. First wash with 350 µl of RW1 is performed centrifuging at 10,000 rpm for 15 s, after the wash a mix compose of 10 µl of DNAse and 70 µl of buffer RDD are added to each column and incubation at room temperature for 15 min is performed, then after incubation another wash with 350 µl of RW1 is performed centrifuging at the same speed and time of the first wash. Buffer RPE (500 µl) is then added to the column and centrifuge for 15 s at 10,000 rpm, the flow through is then discarded. Another wash step with RPE is then performed, centrifuge step this time is for 2 min at the same speed. Then spin column is then placed on a new 2-ml collection tube and 1 min centrifuge at full speed is performed to eliminate RPE buffer carryover.

Finally, to elute the RNA a new 1.5-ml collection tube is positioned under the spin column, and 30 µl are gently posed on the filter and 1 min centrifuge is performed to elute the RNA.

An RNA sequencing (RNA-seq) with a coverage of 30 million reads (paired end) was performed on control samples and on mutant heterozygous *Setd5* clones. RNA was extracted as described below.

The sequencing was performed by the Centre for Translational Genomics and Bioinformatics at San Raffaele Hospital. Libraries have been prepared using Tru Seq RNA v2 kit (Illumina) starting from total RNA. Sequencing was performed using HiSeq 2500 (Illumina).

### RNA sequencing analysis

Fastq files, after adaptors trimming using Trimmomatic [30], were aligned to the 10-mm mouse reference genomes using Bowtie2 [31]. Differential gene expression and Functional enrichment analyses were performed with DESeq2 [32] and GSEA [33], respectively. Statistical and downstream bioinformatics analysis were performed within the R environment. RNA-seq of NSCs cultured in low oxygen condition was performed as described in our previous work [24].

The data were deposited in the NCBI Gene Expression Omnibus and are accessible through GSE103912 (NSCs low oxygen) and GSE222188 (NSCs high oxygen).

### RNA extraction with trizol

To perform RNA extraction, NSCs were firstly seeded in adherent condition using matrigel 1:100 in 6 wells at a density of at least 200,000 per well in NIM.

After 2–3 days, medium was removed, cells were washed once with PBS and were dissolved using 1 ml of Trizol (ThermoFisher Scientific) 5 min at room temperature, directly in the well. After dissolving, samples were collected in 1.5-ml tubes (Eppendorf) and 0.2 ml of chlorophorm were added, tubes were then shaken vigorously and then incubated 2–3 min at room temperature. Tubes were subsequently centrifuged at 13,000 rpm for 15 min at 4°. An aqueous phase appears at the top of the liquid volume, that is collected and transfer to a new tube, the rest is discarded.

To precipitate RNA Isopropyl alcohol is added, 0.5 ml per 1 ml of Tryzol, shake and incubated for 10 min at room temperature before centrifuge 10 min at 13,000 rpm at 4°. After this passage, a pellet is often visible, supernatant is removed and 1 ml of 75% ethanol per ml of Tryzol is added, then samples are vortexed and centrifuged at no more than 7500 rpm for 5 min at 4°. Finally, ethanol is removed, and RNA pellet is dried at room temperature, then a suitable volume of nuclease free water is added, and samples are posed at 55° for a maximum time of 10 min to allow complete resuspension of RNA.

### Retrotranscription and real-time quantitative PCR

RNA retrotranscription was performed using ImProm II reverse transcription kit (Promega).

Briefly, the reaction is divided in two parts. The first reaction is a denaturation step of a mix composed of 1 µg of RNA, random primers (0.5 μg/reaction) and water up to 5 μl, for 5′ at 70 °C. Then after chilling the first mix on ice for 5′, a second retronscription mix (4 μl of ImProm II 5 × buffer, 3.8 μl MgCl2 (final concentration 2.5 mM), 1 μl dNTP Mix (final concentration 0.5 mM each dNTP), 1 μl ImProm II reverse transcriptase, 5.2 of nuclease free water) is added to first mix, and then reverse transcription is performed with the following reaction profile:

1. 4° × 30″
2. 25° × 5′
3. 42 × 1 h
4. 70° × 15′
5. 16 × ∞

After c-DNA synthesis real-time quantitative PCR was performed (RT-qPCR) using CFX 96 Real-Time System (Biorad) thermocycler.

The real-time TITAN (Bioatlas) mix was composed as follows for each reaction samples:

- 2 μl of c-DNA
- 9.8 μl of H2O
- 3.2 μl of TITAN HotTaq EvaGreen qPCR Mix (Titan hotTaq DNA polymerase, 5 × qPCR buffer E, 12.5 mM MgCl2)

The amplification profile used was the following:

- 95° × 15′
- (95° × 15″, 60° × 20″, 72° × 20″) × 40
- 65°

RT-qPCR data were analyzed using CFX Manager software (Biorad) and the differential gene expression was calculated using the ∆∆ct methods. Used primers are listed in the Additional file 3: Table S3.

### Mitochondrial fragmentation analysis in NSCs

NSCs were plated on Matrigel-coated glass coverslips (13 mm) and were fixed for 20 min on ice in 4% paraformaldehyde (PFA, Sigma), solution in phosphate-buffered saline (PBS, Euroclone). Two washes were performed afterwards with PBS, cells were then permeabilized for 30 min in blocking solution, containing 0.1% Triton X-100 (Sigma-Aldrich) and 5% donkey serum (Sigma-Aldrich), and incubated overnight at 4 °C with antibody against transporter of outer mitochondrial membrane 20 (TOMM-20) diluted in blocking solution. The next day, cells were washed 3 times with PBS for 5 min and incubated for 1 h at room temperature with Hoechst 33,342 (ThermoFischer Scientific) and specific secondary antibodies (ThermoFisher Scientific) in blocking solution.

Images were acquired using confocal microscope (Leica Sp5). Fragmentation level was assessed based on circularity calculated using ImageJ plugin mitochondrial morphology (http://imagejdocu.tudor.lu/doku.php?id=plugin:morphology:mitochondrial_morphology_macro_plug-in:start).

### Electron microscopy

Transmission electron microscopy (TEM) was performed at Advanced Light and Electron Microscopy BioImaging Center (ALEMBIC) at San Raffaele Hospital. One NSC WT sample and one *Setd5* heterozygous NSCs sample were cultured in adherent condition in 10 mm Petrie dish till reaching 90% of confluence. Cells were then pelleted at 1200 rpm for 5′ and then included in TEM fixative solution for further analysis.

For adult mice, the brain of wild type and *Setd5*+*/*− were extracted from the skull after brief perfusion with Sodium Chloride 0.9% (S.A.L.F) and then included in TEM fixative solution for further analysis.

Major axis of mitochondria was measured using ImageJ software. Qualitative evaluation of mitochondria was performed manually on each image.

### Western blot

To perform Western Blot analysis cells were seeded in adherent condition in 15 mm Petrie dish till 90% of confluence. Cells were lysed in RIPA buffer (50 mM Tris–Hcl ph 7.4, 150 mM NaCl, 0.1% SDS, 1% Triton X-100, 2% complete protease inhibitor cocktail (Roche), 10%complete phosphatase inhibitor cocktail) for 30 min at 4°. After the lysis samples were centrifuged at 13,000 rpm for 30 min and the supernatant was rescued. Protein samples were then quantified using BCA protein assay kit (ThermoFisher).

After quantification protein samples containing 30 µg of protein, sample buffer (Tris–Hcl ph 6.8 150 mM, glycerol 45%, SDS 7,50%, Bromophenol blue 0.08%) and H_2_O up to 24 µl each were prepared and boiled for 5 min at 95°.

Samples were loaded on poly-acrylamide gel and after proper run were transferred on nitrocellulose membranes for 90–120 min at constant intensity of 350 mA.

Blocking reaction was performed using 5% milk in PBS-T (PBS with 0.1% tween) for 1 h at room temperature. Incubation with primary antibody (Additional file 3: Table S3) was performed over/night at 4° in blocking solution at the proper dilution. The next day membranes were washed 3 times, for 15–20 min in PBS-T and incubation with secondary antibody (Additional file 3: Table S3) conjugated with horseradish peroxidase (HRP) was performed at the proper dilution in blocking solution for 1 h at room temperature.

Finally, to reveal the signal ECL prime kit (GE healthcare) has been used and images were acquired using a chemiluminescent reader.

Densitometry was calculated using ImageLab software (Biorad).

### MitoTracker Orange and MitoTracker Green FM cellular staining

NSCs were analyzed by fluorescence activating cell sorter (FACS) using LSRFortessa analyzer (BD Bioscience), using two different fluorescent dyes, MitoTracker^®^ Green FM (ThermoFisher) and MitoTracker^®^ Orange CMTMRos (ThermoFisher). The first one stains mitochondria regardless of the membrane potential, the second one stains mitochondria depending on the membrane potential (Fig. 2). Using both at the same time allows to determine if there is a difference in the membrane potential and in the total mitochondria mass in the whole cell population.

Cells were seeded at a density of approximately 500,000 cells per well in 6 wells in adherent condition the day before the analysis. The next day cells were stained with MitoTracker Green FM (stock 1 mM in DMSO) and MitoTracker Orange CMTMRos (stock 1 mM in DMSO) at a final concentration of 50 nM diluted in NIM in both cases. Each sample was single and double stained with both dyes.

### Measurement of membrane potential using TMRM

To perform live imaging on NSCs with TMRM, cells were seeded in adherent condition on glass coverslips at a density of 30,000 cells per well in 24 wells plate. The die was diluted at a final concentration of 250 nM and then cells were incubated at 37° for 10′ or 20′ before image acquisition.

To measure the maximum level of membrane potential cells were treated with complex V inhibitor oligomycin at concentration of 1 µM. To measure the background level of TMRM fluorescence, NSCs were treated with decoupling agent FCCP at concentration of 0.7 µM.

After incubation with the fluorescent dyes, coverslips were mounted on cover glasses using mounting medium, without performing fixation and images were immediately acquired using epifluorescence microscopy. Images were quantified and analyzed using ImageJ software, using gray scale to quantify the fluorescence intensity of the cells for TMRM.

### Cells ATP concentration analysis

Cellular ATP concentration was determined using ATP-Lite assay kit (Perkin-Elmer), an assay that use luminescence to determine cellular ATP concentration.

Cells were seeded in 96 wells plate coated with Matrigel 24 h before the experiment at a density of 30,000 cells per well. The next day cells were lysed using the Mammalian cells lysis solution provided by the kit, then the plate is shaken at 700 rpm for 5 min. The lysates are then transferred to a dark adapt plate and the substrate solution is added and the plate is shaken for 5 min avoiding light exposure, then after ten minutes the plate can be read using a luminometer.

### Lactate medium concentration determination

Lactate concentration in the medium were measured EnzyChrom™ L-Lactate Assay Kit. Cells were seeded in a Matrigel-coated 96 wells plate at a density of 30,000 cells per well. After 72 h 20 µl of medium are taken for each well and 80 µl (60 µl of assay buffer, 1 µl of Enzyme A, 1 µl of Enzyme B, 10 µl of NAD and 14 µl of MTT). Optical density (OD) is read at 565 nm at time 0 and at time 20, 20 min after adding the working solution. OD values obtained at time 0 are subtracted to time OD obtained at time 20 and it’s possible to calculate the lactate concentration building a standard curve using, the range of detection varies depending on the medium or samples that is measure. For cell cultured in phenol red containing media, the range of concentrations detectable spans from 0.1 to 1 mM.

### NAD/NADH cell concentration measurement

To quantify the total intracellular concentration of NAD/NADH, NSCs were seeded in a Matrigel-coated 96 wells plate at a density of 30,000 cells per well 24 h before the experiment.

The NAD/NADH-Glo™ Assay (Promega) kit was used according to the manufacturer’s instructions.

### Measurement of oxidative stress with DCF and DHE

To perform live imaging on NSCs with H2DCFDA and DHE, cells were seeded in adherent condition on glass coverslips at a density of 30,000 cells per well in 24 wells plate. Both dyes were diluted in PBS at a concentration of 10 µM for H2DCFDA and 3.2 µM for DHE, together with Hoechst to stain nuclei. NSCs were then incubated 15′ for H2DCFDA and for 10′ for DHE at 37°. After incubation with the fluorescent dyes, coverslips were mounted on cover glasses using mounting medium, without performing fixation and images were immediately acquired using epifluorescence microscopy. Images were quantified and analyzed using ImageJ software, using gray scale to quantify the fluorescence intensity of the cells for H2DCFDA, or the nuclei for DHE.

### Mitochondria analysis in cortical neurons using mito-dsRed

Cortical Neurons were obtained by dissecting telencephalic cortex of embryos, wild type and *Setd5*+*/*−, at day 17.5 post-conception from wild type and Setd5+*/*−. Briefly, after dissection, the cortex was digested 30′ with trypsin. After 3 washes with HBSS medium, the tissue was gently dissociated mechanically by using pipette, DNAse was added to avoid cell clumping and cells were centrifuged 5′ at 1200 rpm. Subsequently, cells were plated at density of 400,000 in an Ibidi, µ-Dish 35 mm, high chamber for live imaging coated the day before with poly-L lysine. The next day cells were infected with CMV-mito-dsRed lentivirus. After 7 days in culture, live imaging was performed using the in vivo imaging setup of Leica sp8 confocal microscope.

For Mdvi-1 rescue experiment, the compound was resuspended in DMSO as vehicle and added directly to the culture media at final concentration of 25 um the day after plating. The treatment was maintained throughout the whole culture period before performing image acquisition.

Mitochondrial fragmentation level was assessed based on circularity calculated using ImageJ plugin mitochondrial morphology (http://imagejdocu.tudor.lu/doku.php?id=plugin:morphology:mitochondrial_morphology_macro_plug-in:start). Other measures were performed using image J software.

### In vivo ATP quantification

Mitochondria were isolated as previously published [34, 35], from the cortex of 3 month old wild-type and Setd5+*/*− mice. In brief, mice cortex was homogenized in an appropriate isotonic buffer [0.25 m sucrose, 20 mm 3-(*N*-morpholino) propane sulfonic acid (MOPS), pH 7.2, 1 mm EDTA, 0.1% BSA fatty acid free and digitonin 0.1 mg/ml] using a glass-Teflon homogenizer. Cell debris and nuclei were pelleted twice by centrifugation at 2500 g for 10 min at 4 °C. Supernatants were centrifuged at 12,500 g for 30 min at 4 °C and the mitochondrial pellet was resuspended in an isotonic buffer (0.5 m sucrose, 20 mm MOPS, pH 7.2, 1 mm EDTA). Isolated mitochondria were incubated at 37 °C for 30 min in a respiratory buffer (0.25 m sucrose, 20 mm MOPS, 1 mm EDTA, 5 mm inorganic phosphate, 0.1% BSA fatty acid free, and 1 mm ADP, pH 7.4) containing specific substrates of the respiratory chain complexes. By providing pyruvate/malate (5 and 1 mm, respectively) and glutamate/malate (5 and 1 mm, respectively), we stimulated ATP synthesis. ATP production was measured by luminometric assay.

### Synaptosomes preparation

Synaptosomes were isolated from cortical brain tissue of *Setd5*^+/+^ and *Setd5*^+/−^ by using the Syn-PER reagent (Thermo Fisher). Briefly, the cortical tissue was isolated and disaggregated into a glass Dounce homogenizer filled up with 4 mL of Syn-PER reagent. After 15 gently strokes, the homogenate was poured in a 15 mL falcon and centrifuged at 1200× *g* for 10 m at 4 °C. The supernatant was then centrifuged at 15,000× *g* for 20 m at 4 °C. The pellet containing the purified synaptosome fraction was weighted for subsequent treatments.

### Statistical analysis

All statistical analyses were performed using *GraphPad PRISM 8.0.2* software, calculating for each sample the arithmetic mean and error, calculated as standard error mean (SEM). In experiments with only two test items (TI), statistical significance was calculated using unpaired T-Test or Mann–Whitney test were indicated.

In experiments with more than two TI, one-way analysis of variance (ANOVA) and Dunn multiple comparison test was performed to determine the statistical significance of the experiments.

In experiments with more than one factor analyze and more than two TI, two-way analysis of variance (ANOVA) and Sidak, Bonferroni or Tuckey multiple comparison test was performed to determine the statistical significance of the experiments.

## Results

### SETD5 controls the transcription of nuclear-encoded mitochondrial genes

**Fig. 1:**
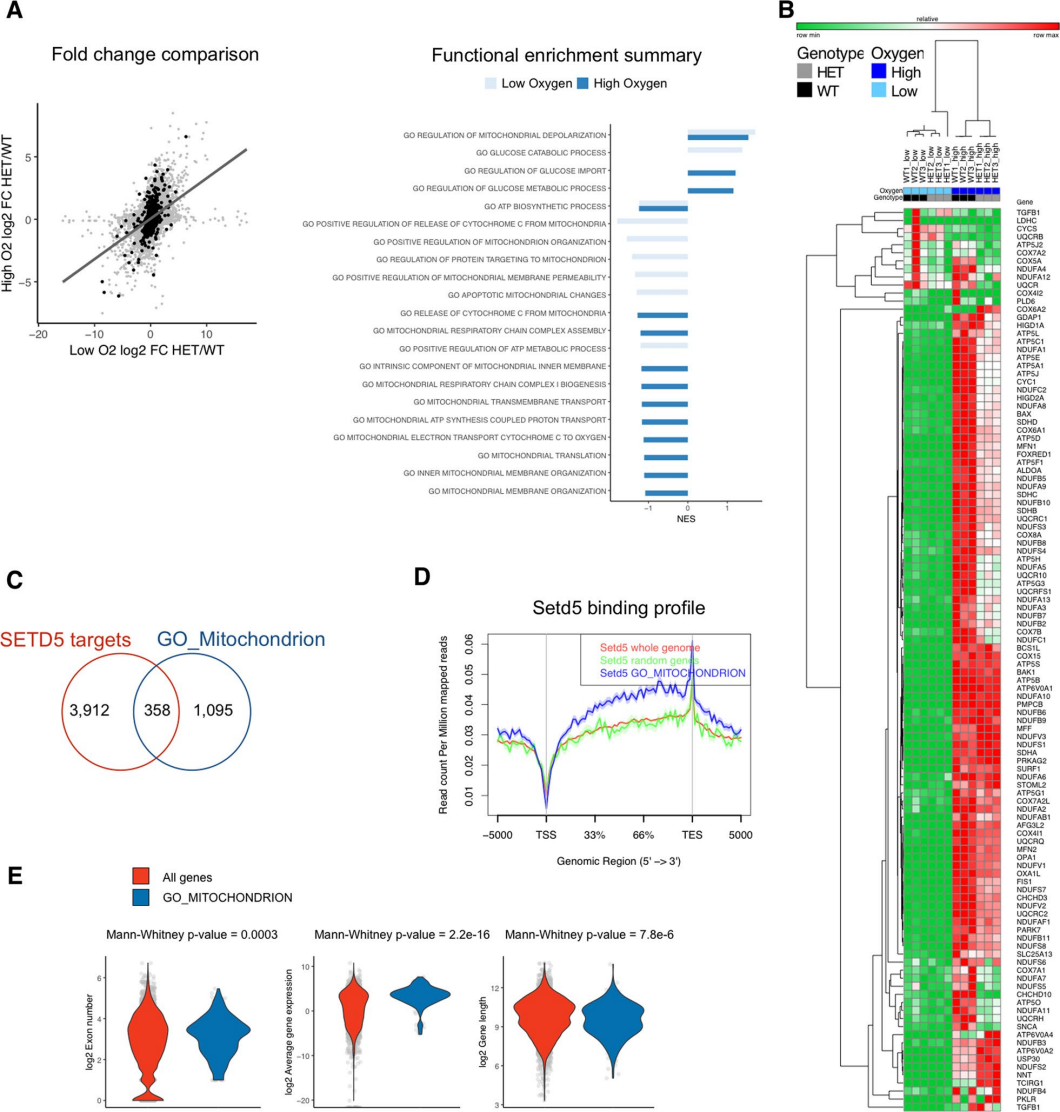
Transcriptional impairment of mitochondrial genes in *Setd5*^+/−^ NSCs. **A** Left, scatter plot showing the comparison of gene transcription fold change (by RNA-seq) between *Setd5*^+/−^ (HET) and control (WT) NSCs cultured at low (5%) and high oxygen (21%) level. Black dots represent mitochondrial genes (belonging to relevant Gene Ontology categories), while gray ones are unrelated genes. Functional enrichment results of differentially regulated genes between HET and WT NSCs in either low (light blue) and high oxygen (blue) condition. Gene Ontology categories related to mitochondrial function and glycolytic pathways are shown. NES, normalized enrichment score. **B** Heatmap showing mitochondrial-related gene expression in HET (gray) and WT (black) NSCs in either low (light blue) and high oxygen (blue) condition. **C** Venn diagram showing the overlap between SETD5 target as previously identified (Sessa et al. 2019) and mitochondrial genes. **D** Density plot for SETD5 ChIP-seq signals on the gene bodies (± 5 Kb) on either all genes (red track), or mitochondrial genes only (blue), or a randomly chosen gene set of the same size (green). **E** Violin plots showing the comparison between all (red) and mitochondrial genes (blue) of exon number, average gene expression and gene length. The mean value plus/minus the standard deviation is plotted in the violin plot. Statistics, Mann–Whitney test

Murine *Setd5* haploinsufficient neural stem cells (NSCs) normally cultured at 5% O_2_, to respect the condition found in the embryo and stem cells niches, showed wide downregulation of mitochondrial-associated genes as well as the increase of response of hypoxia including glycolysis by RNA-seq (Additional file 1: Table S1, Additional file 4: Fig. S1A, B) [24]. We reasoned that culturing the NSCs upon ambient air condition (21% O_2_) possibly uncover mitochondrial impairment in Setd5 haploinsufficiency (Additional file 4: Fig. S1C). We found that high oxygen further exacerbated the deregulation of the mitochondrial gene subset (Fig. 1A, Additional file 1: Table S1, Additional file 4: Fig. S1D, E) being the mutant cells unable to properly react to the oxygen increase, at least at the transcriptional level (Fig. 1B). Interestingly, ~ 30% of the mitochondrial-associated genes were among the SETD5 target genes identified by ChIP-seq in the same cells (Fig. 1C, Additional file 2: Table S2) [24]. Of note, the SETD5 binding on gene bodies was particularly relevant on mitochondrial genes compared to both all genes and a random subset of the same size as indicated by the density plot (Fig. 1D, Additional file 2: Table S2). In accordance with the showed SETD5 binding preference, the mitochondrial subset encloses genes that are both rich in exons, highly expressed and long, features that emerged as correlated with a high probability of SETD5 genomic association (Fig. 1E) [24].

Together these observations highlight the importance of correct levels of SETD5 to promote the transcription of mitochondrial-associated genes especially when those genes are specifically required, i.e., in response to high O_2_ tension.

### *Setd5* haploinsufficient NSCs show impaired mitochondria

**Fig. 2:**
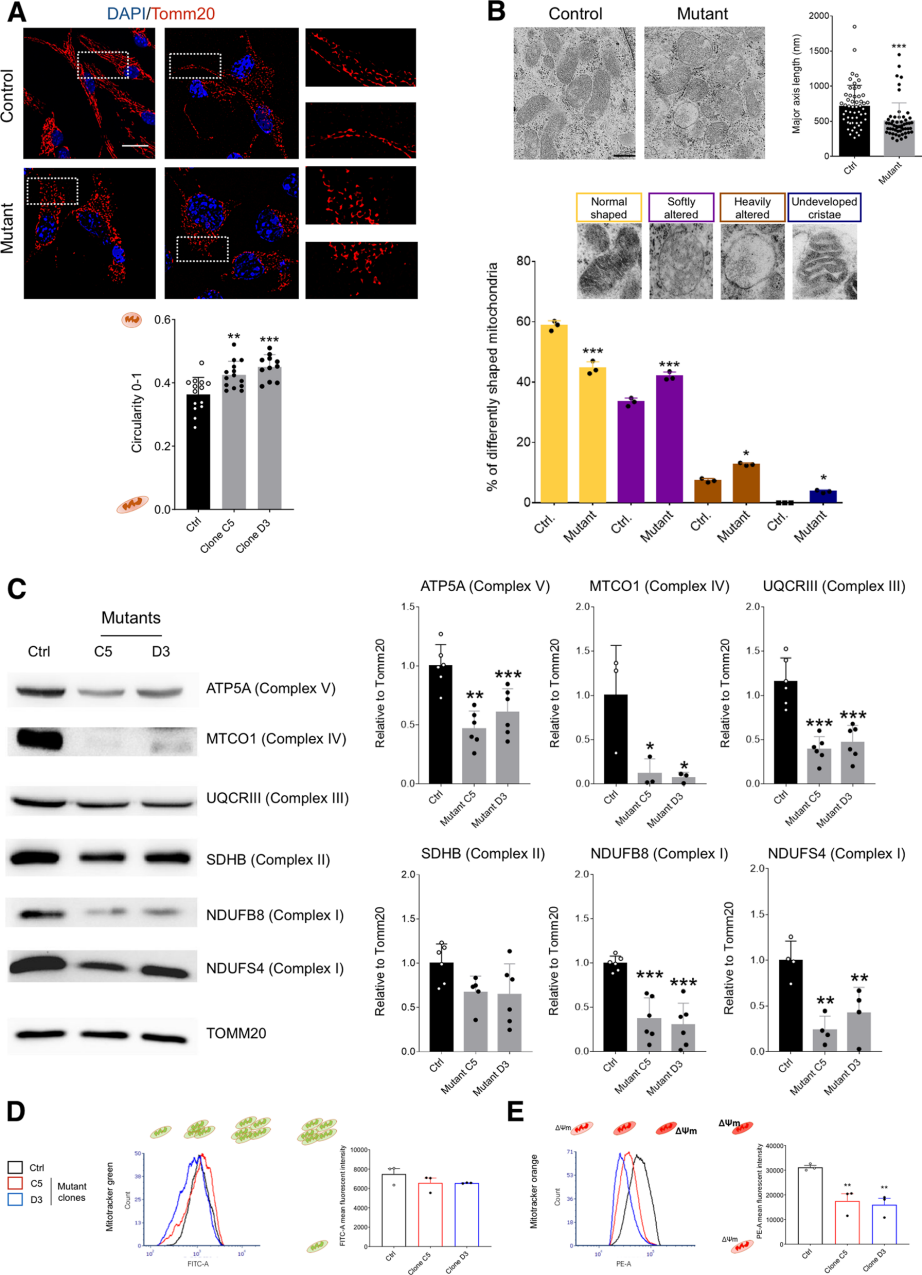
Mitochondrial damage in *Setd5*^+/−^ NSCs. **A** Control and mutant NSCs (two different clones, C5 and D3) immuno-stained with TOMM-20 mitochondrial marker. Nuclei are stained with Hoechst 33,342. dashed rectangles identify the magnification shown on the right (scale bar = 10 micron). The level of circularity, as calculated by ImageJ macro (see methods) between 0–1 is shown. Statistics, one-way ANOVA (*P* = 0.0001), multiple comparison Dunnet test (Ctrl vs. Clone C5, *P* = 0.0023; Ctrl vs. Clone D3, *P* < 0.0001) **B** Top, ultrastructural analysis by transmission electronic microscopy (TEM) of mitochondria in *Setd5*^+/−^ (Mutant) and control (WT) NSCs (scale bar = 500 nm). Quantification of the mitochondria major axis length is shown on the right. Statistics, Mann–Whitney test (*P* < 0.0001). Bottom, quantification of the different kind of mitochondria retrieved as indicated. Statistics, one-way ANOVA (*P* = 0.0001), multiple comparison Tuckey test (Ctrl vs. Mutant, *P* < 0.0001; Ctrl vs. Mutant, *P* < 0.0001; Ctrl. vs. Mutant, *P* = 0.0325; Ctrl vs. Mutant, *P* = 0.0257). **C** Western blot analysis on whole protein lysate from control and *Setd5*^+/−^ NSCs (two different clones, C5 and D3) for the indicated mitochondrial protein, normalized on total Tomm-20 level. Relative quantification on the right. Statistics; NDUFS4 (Complex I) one-way ANOVA (*P* = 0.0002), multiple comparison Dunnet test (Ctrl vs. Clone C5, *P* = 0.0006; Ctrl vs. Clone D3, *P* < 0.0002); NDUFB8 (Complex I), one-way ANOVA (*P* = 0.0002), multiple comparison Dunnet test (Ctrl vs. Clone C5, *P* = 0.0006; Ctrl vs. Clone D3, *P* < 0.0002); SDHB (Complex II), one-way ANOVA (*P* = 0.0672), multiple comparison Dunnet test (Ctrl vs. Clone C5, *P* = 0.1025; Ctrl vs. Clone D3, *P* = 0.0647); UQCRIII (Complex III), one-way ANOVA (*P* < 0.0001), multiple comparison Dunnet test (Ctrl vs. Clone C5, *P* < 0.0001; Ctrl vs. Clone D3, *P* < 0.0001); MTCO1 (Complex IV), one-way ANOVA (*P* = 0.0265) multiple comparison Dunnet test (Ctrl vs. Clone C5, *P* < 0.0338; Ctrl vs. Clone D3, *P* < 0.0278); ATP5A (Complex V) ANOVA (*P* = 0.0003) multiple comparison Dunnet test (Ctrl vs. Clone C5, *P* = 0.0002; Ctrl vs. Clone D3, *P* = 0.0032). **D**, **E** Both control and mutant NSCs (three different clones, B4, C5, and D3) were treated with cell-permeant fluorescent dyes that are either insensitive (Mitotracker green, **D**) or sensitive (Mitotracker orange, **E**) to mitochondrial membrane potential. Flow cytometry analysis estimate either mitochondrial mass using mitotracker green (**D**) or mitochondrial potential with the orange (**E**). Mitotracker Green. Statistics, one-way ANOVA (*P* = 0.3081), multiple comparison Dunnet (Ctrl vs. Clone C5, *P* = 0.3142; Ctrl vs. Clone D3, *P* < 0.3051). Mitotracker Orange. Statistics, one-way ANOVA (*P* = 0.0068), multiple comparison Dunnet (Ctrl vs. Clone C5, *P* = 0.0100; Ctrl vs. Clone D3, *P* < 0.0067. All Data are presented as mean values+*/*− SEM

Since the observed transcriptional deregulation, we investigated the mitochondrial compartment in both wild-type (WT) and *Setd5* mutant NSCs upon high O_2_ levels. Initially, we evaluated the fusion/fission dynamic through both immunocytochemistry and electron microscopy. TOMM20 immunostaining indicated that mitochondria in *Setd5* mutant NSCs were more fragmented compared to the WT (Fig. 2A) while transmission electron microscopy confirmed the smaller size of mitochondria and showed the altered ultrastructural morphology with a high number of organelles with aberrant cristae structure (Fig. 2B). Interestingly, among genes downregulated in our RNA-seq dataset, we found genes encoding for important regulators of mitochondrial dynamics (*Mfn1* and *Opa1*) and biogenesis (*Pgc1α*,* Polg*) that indeed were decreased in mutant cells compared to the WT (Additional files 4, 5: Figs. S1C, E, S2A).

**Fig. 3:**
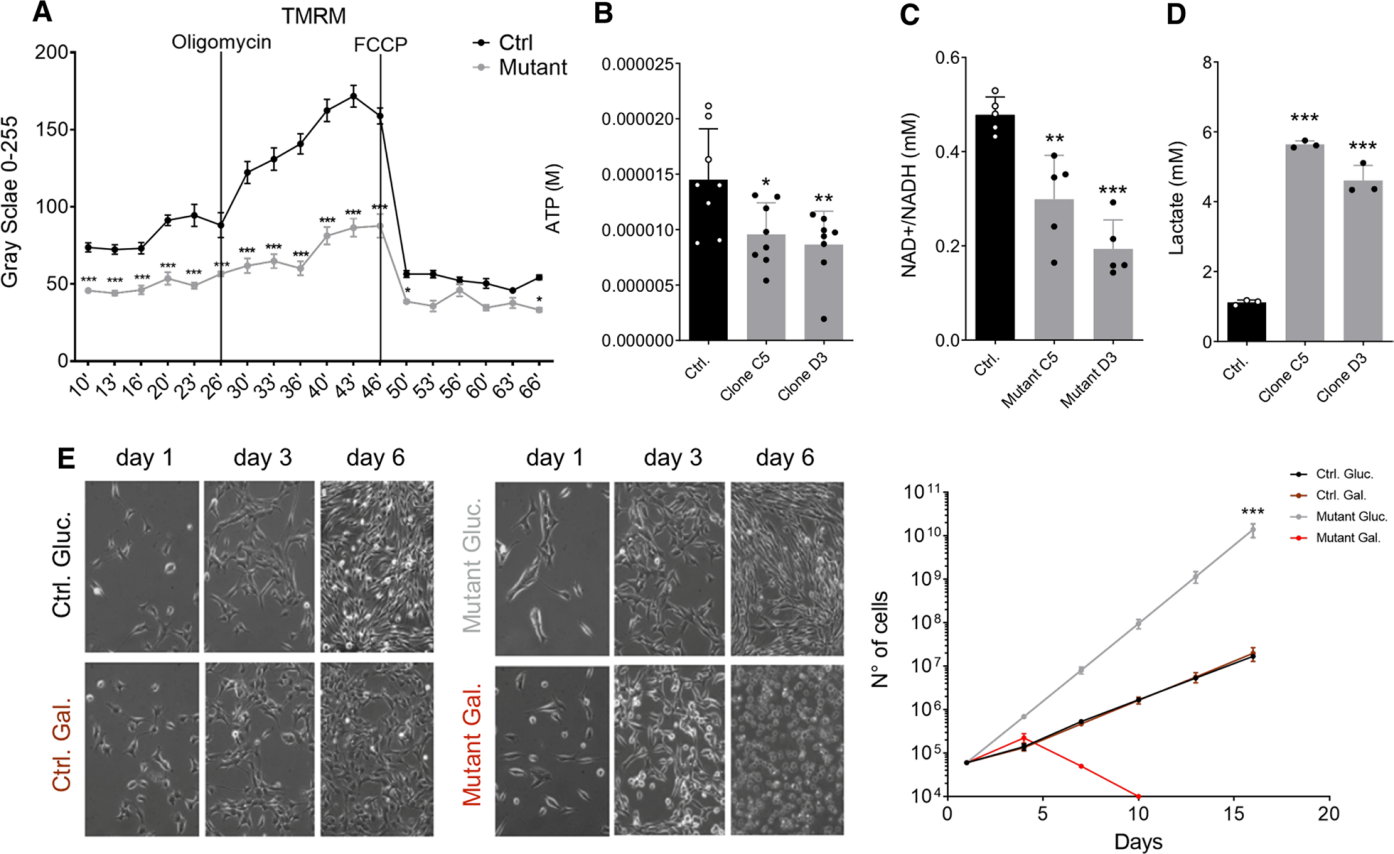
Impaired mitochondrial functionality in *Setd5*^±^ NSCs. **A** Live imaging quantification of TMRM signals in both control and mutant NSCs during the time course of the experiment. Oligomycin and FCCP drugs were added at the indicated time points. Statistics, two-way ANOVA (*P* < 0.0001, for time, interaction and column factor), multiple comparison Bonferroni (Ctrl vs. Mut, 10′, *P* = 0.0003; 13, *P* = 0.0002; 16′, *P* = 0.0006; 20′, *P* < 0.0001; 23′, *P* < 0.0001; 26′, *P* < 0.0001; 30′, *P* < 0.0001; 33′, *P* < 0.0001; 36′, *P* < 0.0001; 40′, *P* < 0.0001; 46′, *P* < 0.0001; 50′, *P* = 0.1020; 53′, *P* = 0.0240; 56′, *P* > 0.9999; 60′, *P* = 0.2509; 63′, *P* > 0.9999; 66′, *P* = 0.0201) **B**–**D** Quantification by fluorescence of the ATP content (**B**), NAD + /NADH (**C**), and lactate (**D**) content in culture of both control and *Setd5*^+/−^ NSCs (two different clones, C5 and D3). Statistics, ATP, one-way ANOVA (*P* = 0.077), multiple comparison Dunnet test (Ctrl vs. Clone C5, *P* = 0.0221; Ctrl vs. Clone D3, *P* = 0.0069). NAD/NADH**,** one-way ANOVA (*P* < 0.0001), multiple comparison Dunnet test (Ctrl vs. Clone C5, *P* = 0.0025; Ctrl vs. Clone D3, *P* < 0.0001). Lactate, one-way ANOVA (*P* < 0.0001), multiple comparison Dunnet test (Ctrl vs. Clone C5, *P* < 0.0001; Ctrl vs. Clone D3, *P* < 0.0001). **E** Bright-field images and growth curves of both control and mutant NSCs cultured either in presence of glucose in the media or without glucose and in presence of galactose as carbon source. Statistics, two-way ANOVA (interaction, *P* < 0.0001, time, *P* < 0.0001, column factor, *P* = 0.0069), multiple comparison Tuckey test (all non-significant, except, Ctrl. Gluc. vs. Mutant Gluc., *P* < 0.0001; Ctrl. Gal. vs. Mutant Gluc., *P* < 0.0001; Mutant Gluc. vs. Mutant Gal., *P* < 0.0001). All Data are presented as mean values+*/*− SEM

Then, we evaluated the amount of several mitochondrial proteins as a proxy of the complexes of oxidative phosphorylation (OXPHOS), also known as the electron transport chain, which is the biochemical core of the mitochondrial functionality. In line with our transcriptional (Fig. 1) and structural data (Fig. 2A, B), *Setd5* mutant mitochondria were deficient in many protein subunits of the different complexes (Fig. 2C). Since the OXPHOS complexes I, II, and IV are proton pumps, we sought defects in the generation of mitochondrial membrane potential (ΔΨm) in mutant cells. Using cell-permeant fluorescent dyes that are either insensitive (Mitotracker green) or sensitive (Mitotracker orange and TMRM) to mitochondrial membrane potential we showed, as expected, lower ΔΨm in *Setd5* mutant cells compared to control despite the total mitochondrial masses were equal (Fig. 2D, E, Additional file 5: Fig. S2B). Mitochondrial membrane potential (measured as Tetramethylrhodamine, Methyl Ester, Perchlorate (TMRM) signal) was then challenged by adding to the medium chemical compounds to induce either hyper- (using oligomycin) or de-polarization (carbonylcyanide-p-trifluoromethoxyphenylhydrazone (FCCP)) [36]. Interestingly, the mutant cells were not able to properly increase their potential, having only a mild response to oligomycin while the uncoupling between the respiratory chain and phosphorylation system due to FCCP was effective (Fig. 3A, Additional file 6: Fig. S3A). Low ΔΨm and blunt reaction to the hyperpolarizing agent would suggest an impairment to generate ATP by OXPHOS. We measured a decrease in total ATP content in mutant cells compared to the control, supporting an energetic defect due to *Setd5* haploinsufficiency (Fig. 3B). Accordingly, the levels of nicotinamide adenine dinucleotide (NAD) were decreased (Fig. 3C). However, the mitochondria of mutated cells seemed not to experience electron leakiness since the evaluation of reactive oxygen species (ROS) resulted in diminished in the mutant cells (Additional file 6: Fig. S3B, C).

We hypothesized that low SETD5 levels may hamper the mitochondrial maturation, and thus the related energy supply, forcing the cells to use glycolysis to survive, as the transcriptional profile already suggested (Fig. 1, Additional file 4: Fig. S1). To investigate this issue, we measured the lactate, a byproduct of glycolysis, that was indeed increased in mutant NSCs (Fig. 3D). To further analyze this aspect, we have grown both WT and mutant NSCs in a culture medium in which the glucose was replaced by galactose as the carbon source for metabolic reaction; the galactose can enter in the glycolytic pathway but without a net gain of energy; moreover, it cannot enter in the pyruvate-lactate anaerobic pathway. Thus, forcing the cells to rely only on mitochondrial respiration, to produce ATP [37]. WT NSCs behaved similarly in glucose and galactose media while the mutant cells, that with glucose proliferated more than the WT, tended to die (Fig. 3E).

These data indicate that low SETD5 levels lead to metabolic reprogramming due to an inefficient mitochondrial dynamic, maturation, and function.

### *Setd5* haploinsufficiency affects mitochondrial machinery and localization in neurons

**Fig. 4:**
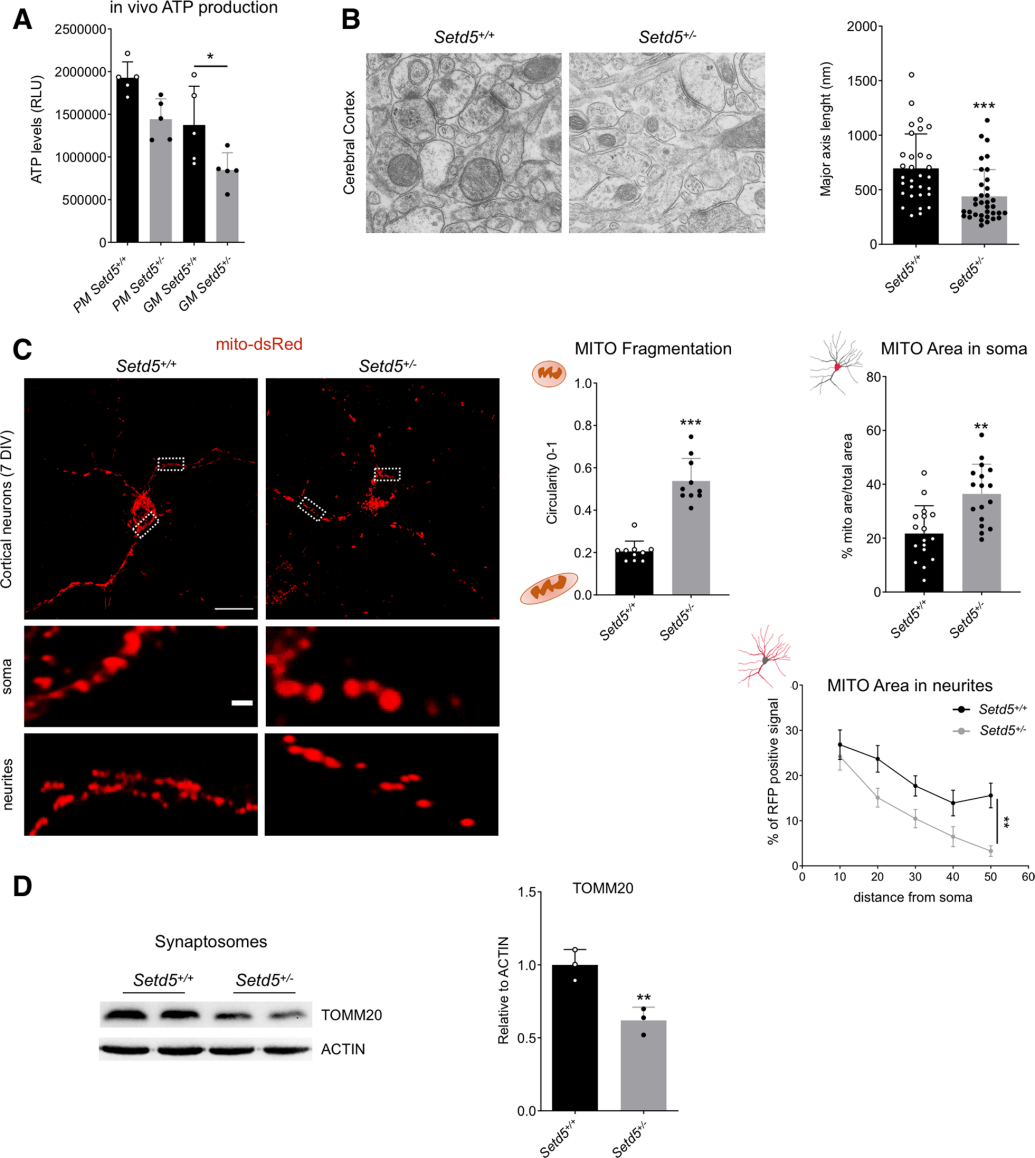
Mitochondrial structure and function in *Setd5*+*/*− mice. **A** In vivo ATP production from isolated mitochondria from the cortex. PM (pyruvate/malate substrate), GM (glutamate/malate). Statistics, PM, Tuckey test, (*Setd5*^+/+^ vs. *Setd5*^+/−^, *P* = 0.0791). GM, Tuckey test, (*Setd5*^+/+^ vs. *Setd5*^+/−^, *P* = 0.05). **B** Ultrastructural analysis by transmission electronic microscopy (TEM) of mitochondria in *Setd5*^+/+^ and *Setd5*^+/−^ mouse cortex (scale bar = 500 nm). Quantification of the mitochondria major axis length is shown on the right. Statistics, Mann − Whitney test (*Setd5*^+/+^ vs. *Setd5*^+/−^, *P* < 0.0001). **C** Cortical Neurons mitochondrial fragmentation and distribution analysis. Mito-dsRed infected cortical neurons (*Setd5*^+/+^ and *Setd5*^+/−^) live imaging (scale bar = 20 micron, magnification, scale bar = 5 micron). MITO fragmentation, statistics, T-test (*Setd5*^+/+^ vs. *Setd5*^+/−^, *P* < 0.0001). MITO area in soma, statistics, T-test (*Setd5*^+/+^ vs. *Setd5*^+/−^, *P* = 0.0003). MITO area in neurites, statistics, two-way ANOVA (row factor, *P* < 0.0001, column factor, *P* = 0.0004, subject, *P* = 0.088), multiple comparison Tuckey test (*Setd5*^+/+^ vs. *Setd5*^+/−^, 10 um, *P* = 0.9554, 20 um, *P* = 0.8030, 30 um, *P* = 0.2020, 40 um, *P* = 0.1782, 50 um, 0.0035) **D** Western blot quantification of mitochondrial fraction in synaptosomes, normalized on actin. Statistics, T-test (*P* = 0.0090)

To better circumstantiate our findings in regard to neurological pathologies associated with *SETD5* mutations, we moved to analyze *Setd5*^+/−^ animal model. We first evaluated the abundance of OXPHOS complexes. In line with NSCs data, we showed that *Setd5*^+/−^ brains presented a decrease in OXPHOS proteins particularly evident in complex I (Additional file 7: Fig. S4A). Evaluation of ATP content in freshly isolated mitochondria from control and *Setd5*^+/−^ brains sustained the in vivo hypo-functionality of oxidative phosphorylation since isolated mitochondria from mutants produced less ATP than their normal counterpart, specifically using glutamate as the substrate for mitochondrial respiration (Fig. 4A).

Using electron microscopy, we retrieved small mitochondria in mutant brains compared to control littermates possibly confirming the defects in fission/fusion dynamics seen in NSCs (Fig. 4B). To better analyze this aspect, we moved to cultured primary neurons. Live staining using the mito-dsRed mitochondrial marker, showed that neurons from E17.5 *Setd5*^+/−^ cortices displayed more fragmented mitochondria compared to controls (Fig. 4C). Interestingly, in mutant neurons, the mitochondria were located primarily in cell soma while were reduced in neurites (Fig. 4C). We reasoned that mitochondria may be stacked in the cell body, with few possibilities to travel along neurites to reach synaptic sites within neurons. To investigate this issue, we isolated synaptosomes containing fraction from *Setd5*^+/+^ and *Setd5*^+/−^ cortices and estimated mitochondria content showing that mutant synapses contained fewer mitochondria (Fig. 4D).

**Fig. 5:**
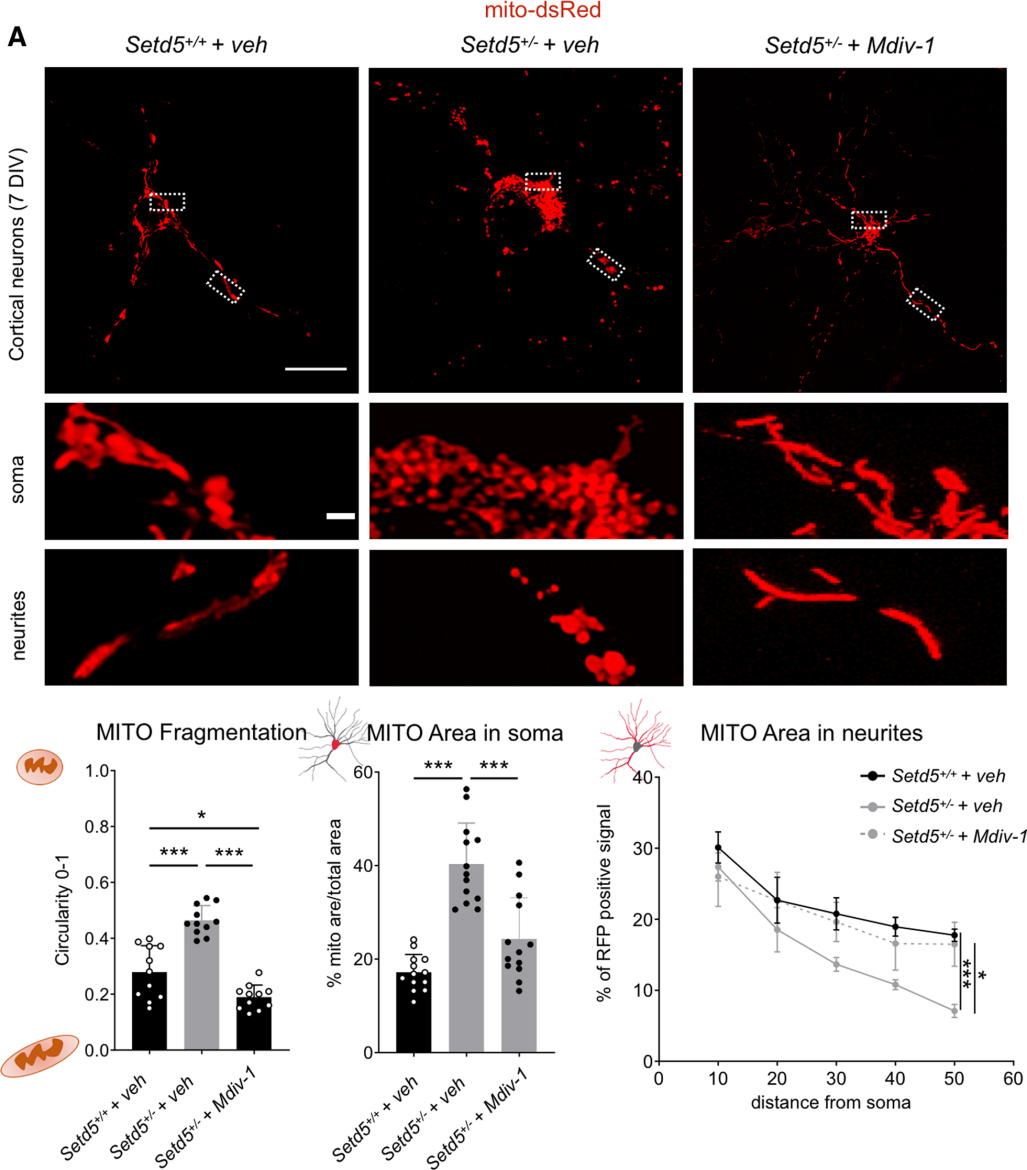
Mdiv-1 treatment. **A** Rescue experiment with *Drp1* inhibitor Mdiv-1. Analysis of mitochondrial fragmentation and distribution in Mito-dsRed infected cortical neurons (*Setd5*^+/+^ and *Setd5*^+/−^, *Setd5*^+/−^ mdvi-1 treated) live imaging (scale bar = 20-micron, magnification, scale bar = 5 micron). Statistics (fragmentation, one-way ANOVA with Tuckey test for multiple comparisons; *Setd5*^+/+^ vs. *Setd5*^+/−^*, P* < 0.0001; *Setd5*^+/+^ vs. *Setd5*^+/−^ Mdvi-1, *P* = 0.0106; *Setd5*^+/−^ vs. *Setd5*^+/−^ mdvi-1, *P* < 0.0001. MITO area in soma, one-way ANOVA with Tuckey test for multiple comparisons; *Setd5*^+/+^ vs. *Setd5*^+/−^, *P* < 0.0001; *Setd5*^+/+^ vs. *Setd5*^+/−^ mdvi-1, *P* = 0.0513; *Setd5*^+/−^ vs. *Setd5*^+/−^ mdvi-1, *P* < 0.0001. MITO area in neurites, statistics, two-way ANOVA (row factor, *P* < 0.0001, column factor, *P* = 0.0008, subject, *P* = 0.51), multiple comparison Tuckey test (*Setd5*^+/+^ vs. *Setd5*^+/−^*,* 10 um, *P* = 0.6279, 20 um, *P* = 0.6306, 30 um, *P* = 0.2004, 40 um, *P* < 0.0001, 50 um, *P* < 0.0001; *Setd5*^+/+^ vs. *Setd5*^+/−^ mdivi-1*,* 10 um, *P* = 0.6698, 20 um, *P* = 0.9999, 30 um, *P* = 0.9465, 40 um, *P* = 0.8264, 50 um, *P* = 0.9159; *Setd5*^+/−^ vs. *Setd5*^+/−^ mdivi-1*,* 10 um, *P* = 0.9547, 20 um, *P* = 0.7055, 30 um, *P* = 0.1319, 40 um, *P* = 0.3155, 50 um, *P* = 0.0261). All Data are presented as mean values+*/*− SEM

The quinazolinone derivative mitochondrial division inhibitor 1 (Mdiv1), reversibly inhibits DRP1, a master regulator of fission machinery [38]. Its action has been linked with increased mitochondrial fusion, attenuation of apoptosis, and cytoprotection [38–42]. We decided to use Mdiv1 in treating *Setd5* mutant neurons. Mdiv1 was effective to increase mitochondrial length in mutant neurons as well as mitochondrial distribution inside the mutant cell soma and neurites (Fig. 5A).

These data indicate that *Setd5* haploinsufficiency in neuronal cells impairs mitochondrial dynamics, functionality, and localization that can be at least in part rescued by the DRP1 inhibitor Mdiv1.

## Discussion

In this study, we revealed mitochondrial dysfunctions as possible contributors to the pathogenesis of neurodevelopmental disorders caused by mutations in the *SETD5* gene. *Setd5* haploinsufficiency in mice is leading to a decrease in mitochondrial membrane potential and subsequent ATP production as well as impairment in organelle dynamics in mutant neuronal cells. Our data suggest that the mutant mitochondria are hypo-functional and mislocalized due to transcriptional defects downstream to low SETD5 activity. Both the consequent metabolic reprogramming of mutant cells and the mitochondrial impoverished neurites and synapses may contribute, at least in part to the pathogenesis of SETD5-associated diseases.

Brain development and related disorders have been associated with mitochondrial functions/dysfunctions [6, 43]. Recent studies have suggested that a large part of ASD patients showed high levels of lactate, pyruvate, and alanine in body fluids and decrease the activity of a variety of mitochondrial respiratory complexes in brain regions as well as in peripheral organs, particularly evident at young age, that eventually were restored in adulthood [44–48]. Interestingly, mutations in the mtDNA have been associated with ASD cases and other NDDs [49, 50]. These observations support the idea that metabolic changes due to mitochondrial impairment may either contribute to or induce those neurodevelopmental defects at the basis of NDDs. For instance, OXPHOS aberrations lead to abnormal neuronal migration [51], while the PolG mutator mouse, a model of mtDNA mutations and respiratory dysfunction due to lack of POLG activity, revealed neural stem cell dysfunctions with decreased capabilities to self-renew and differentiate [52].

Deregulation of the fission/fusion processes is intimately implicated in neurological disorders. The strong association between increased mitochondrial fragmentation and neurodegenerative diseases (e.g., Parkinson’s, Alzheimer’s, and Huntington’s diseases) underlines the importance of mitochondrial dynamics in neuronal function and survival [43]. Moreover, mutations in genes encoding for key regulators of fission/fusion, such as *DRP1*, *MFN1*, *MFN2*, and *OPA1* genes among others, also induce neurodevelopmental defects such as brain abnormalities, decrease the number of neurites and synapses up to neuronal death both in humans and animal models [53–57]. One of the proposed mechanisms at the basis of the pathological onset is the fact that fragmented mitochondria become refracting to movements within polarized cells as neurons, possibly because small organelles may not arrange enough molecular adaptors for transport, as Miro/Milton, for their correct mobility [57, 58]. As consequence, the organelles are not available in highly demanding cellular compartments, such as synapses, causing low ATP supply and Ca^2+^ buffering with functional impairment for the neuron. Accordingly, we report that *Setd5* animal models presented fragmented mitochondria stalled in the cell body that eventually contribute to the loss of synapses and neuronal hypo-functionality that characterize these mice [24, 26].

A recent report proposed that SETD5 regulates glycolysis through the regulation of EP300/HIF-1a co-activators upon the hypoxic condition in breast cancer stem-like cells [59]. In our models, we did not find evidence for such function in regulating glycolytic genes. In fact, glycolysis seemed to sustain the ATP production in *Setd5* haploinsufficient cells. Further investigations are needed to understand whether a direct effect on glycolysis is cell-, environmental-specific, or coupled and eventually masked by mitochondrial impairment.

The interplay between epigenome and mitochondria is bidirectional: mitochondrial are essential for the post-translational histone modifications for instance supplying acetyl-CoA and S-adenosyl methionine (SAM) for acetylation and methylation, and, conversely, epigenetic changes influence mitochondrial biogenesis and function [60]. Compelling examples of the latter are given by: (1) the inhibition of SET containing protein SETD7, a histone methyltransferase to H3K4me1, promotes mitogenesis through activation of PGC1A [61]; (2) the activity of *LSD1,* the first identified H3K4me1/2 demethylase, also regulates mitochondrial activity at different extent depending the system [34, 35, 62–64]; (3) the depletion of H3K4 methylase SMYD1 leads to a reduction in mitochondrial energetics in the heart due to PGC1A repression [65].

## Limitations

One limitation of the study is that we used only a murine experimental setup, while species-specific differences may occur and the phenotype in human cells and organisms be different. Further experiments will clarify this point. Another important issue is that we can only speculate about the possible primary causative role of mitochondrial impairment in the establishment of the SETD5-related NDDs. Indeed, as for other disorders related to brain development that have been associated with mitochondrial damage, it remains challenging to claim that mitochondria-associated phenotypes are the major driver of the diseases. Specifically, for SETD5 its broad impact on gene transcription not related with mitochondria is contributing to the disease, as previously suggested.

## Conclusions

In summary, our data indicate that massive deregulation of transcription, including mitochondrial master regulators such as *Pgc1a* and *Polg*, is at the basis of a variety of mitochondrial aberrations both at the levels of neural precursors and adult neuronal cells which carry *Setd5* haploinsufficiency. These impairments ranging from hypo-functionality of the OXPHOS machinery up to mitochondrial miss-localization and fission alterations. Despite no report or evidences link SETD5 patients with mitochondrial defects, our work suggests that those may contribute to SETD5-related pathologies. In fact, this kind of defects may be overlooked especially if they occur in inaccessible developmental stages. Further work is needed to better determine the extent of the pathology that is properly caused by mitochondrial impairment and, consequently, if the mitochondria dysfunctions should be considered novel therapeutic targets for SETD5-related NDDs.

## Supplementary Information

**Additional file 1**: **Table S1**. RNA-seq normalized counts, functional enrichment data for High & Low Oxygen condition.

**Additional file 2**: **Table S2**. Gene list of SETD5 targets, mitochondrial genes and random dataset used to perform comparison in Fig. 1.

**Additional file 3**: **Table S3**. List of primers and antibodies used in this work.

**Additional file 4**: **Figure S1**. Level of oxygen and mitochondrial genes in Setd5+/− NSCs.Enrichment plots from gene set enrichment analysis for Glycolysis, electron transport chain, Mitochondria and Hypoxia, between Setd5+/−and controlNSCs cultured at lowand high oxygenlevel.Western blot analysis for SETD5 protein level in NSCs cultured at 21% oxygen. Actin was used as loading control.RT-qPCR validation of transcription level of the indicated mitochondrial-related genes between Setd5+/−and controlNSCs cultured at lowand high oxygenlevel. All data are presented as mean values ± SEM.

**Additional file 5**: **Figure S2**. Mitochondrial membrane potential is altered in Setd5+/− NSCs.Western blot quantification of Opa1and Mfn1protein level in NSCs normalized on Tomm-20. Statistics, Opa1, one-way ANOVA, multiple comparison Dunnet test; Mfn1, one-way ANOVA, multiple comparison Dunnet test.Quantification of mitochondrial membrane potential using TMRM in live imaging. The signal level is calculated by performing a ratio between the basal fluorescence intensity and the fluorescence after FCCP treatment. Statistics, one-way ANOVA, multiple comparison Dunnet test. All data are presented as mean values +/− SEM.

**Additional file 6**: **Figure S3**. Mitochondrial function alteration in Setd5+/− NSCs.TMRM live staining on control and Setd5+/− NSCs. From the left, basal condition, oligomycin treatment and last two images FCCP treatment.Reactive oxygen species quantification in control and Setd5+/− NSCs performing live staining of DHEand DCF. Statistics, DHE, one-way ANOVA, multiple comparison Dunnet test; DCF, one-way ANOVA, multiple comparison Dunnet test. All data are presented as mean values +/− SEM.

**Additional file 7**: **Figure S4**. Electron transport chain alteration in mouse cortex.Western blot analysis on whole protein lysate from Setd5+/+ and Setd5+/− mouse cortex for the indicated mitochondrial protein, normalized on total Tomm-20 level. Statistics, NDUFB8, T-test,; SDHB,; UQCRIII,; MTCO1,; ATP5A,. All data are presented as mean values +/− SEM.

**Additional file 8**: **Figure S5**. Uncropped Western blot images.The images refer to Fig. 2C.The images refer to Fig. 4D.The images refer to the figure S1C.The images refer to the figure S2A, B.The images refer to the figure S4A.

## Data Availability

RNA-seq raw data are available at GSE103912 for NSCs cultured in low oxygen condition and GSE222188 for NSCs cultured in high oxygen condition.

## Abbreviations

ASD: Autism spectrum disorder
FCCP: Carbonylcyanide-*p*-trifluoromethoxyphenylhydrazone
ID: Intellectual disability
Mdiv1: Mitochondrial division inhibitor 1
NAD: Nicotinamide adenine dinucleotide
NDD: Neurodevelopmental disorder
NSC: Neural stem cell
OXPHOS: Oxidative phosphorylation
ROS: Reactive oxygen species
SAM: S-adenosyl methionine
TMRM: Tetramethylrhodamine, methyl ester, perchlorate
WT: Wild type
