# Supplementary figures and images for: SETD5 haploinsufficiency affects mitochondrial compartment in neural cells
Source: Mol Autism. 2023 Jun 1;14:20. doi: 10.1186/s13229-023-00550-9 (PMC10233863; doi:10.1186/s13229-023-00550-9)

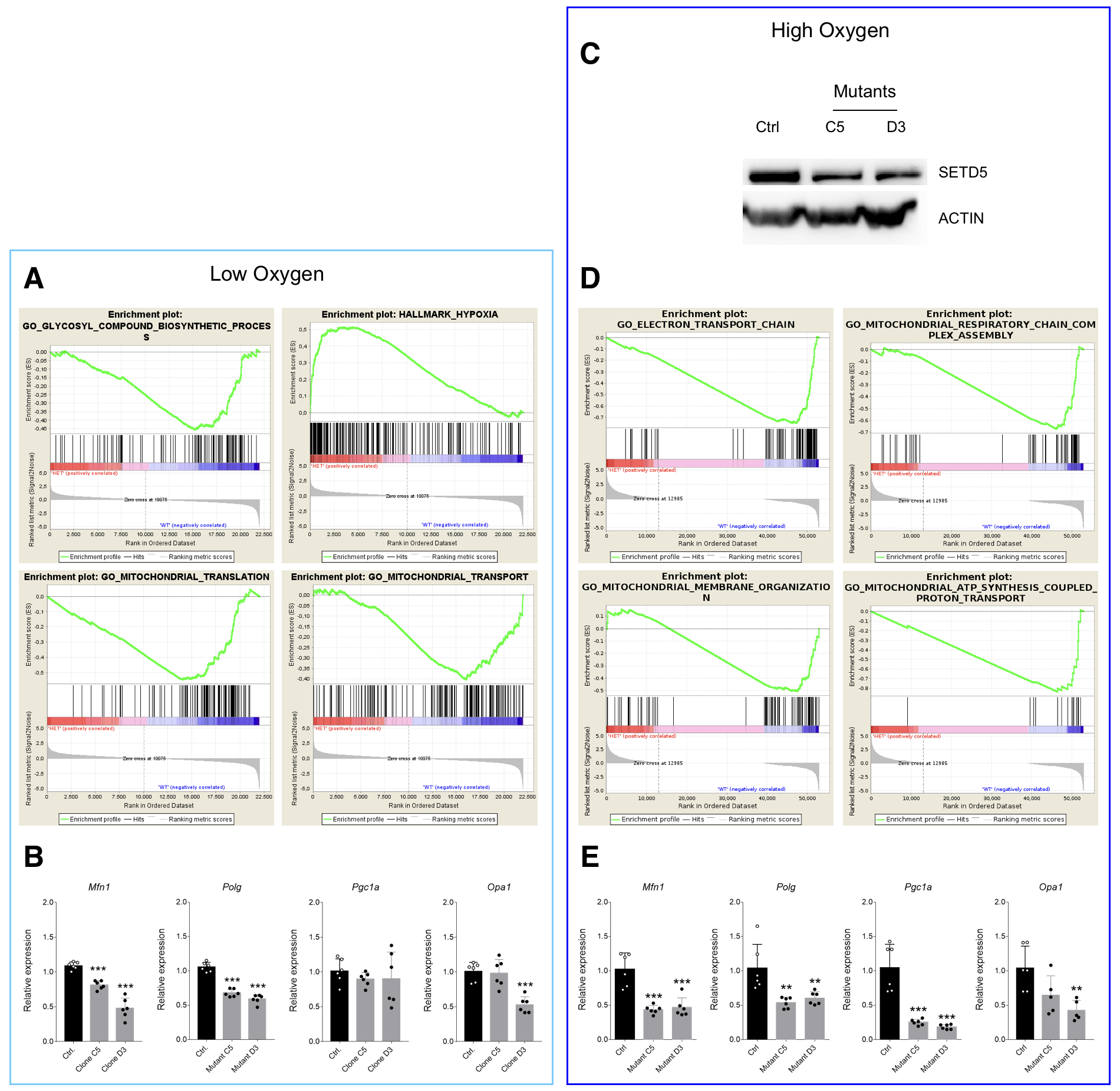

Supplement: Supplementary file 4 — Additional file 4: Figure S1. Level of oxygen and mitochondrial genes in Setd5+/− NSCs.Enrichment plots from gene set enrichment analysis for Glycolysis, electron transport chain, Mitochondria and Hypoxia, between Setd5+/−and controlNSCs cultured at lowand high oxygenlevel.Western blot analysis for SETD5 protein level in NSCs cultured at 21% oxygen. Actin was used as loading control.RT-qPCR validation of transcription level of the indicated mitochondrial-related genes between Setd5+/−and controlNSCs cultured at lowand high oxygenlevel. All data are presented as mean values ± SEM. [file 13229_2023_550_MOESM4_ESM.tif]

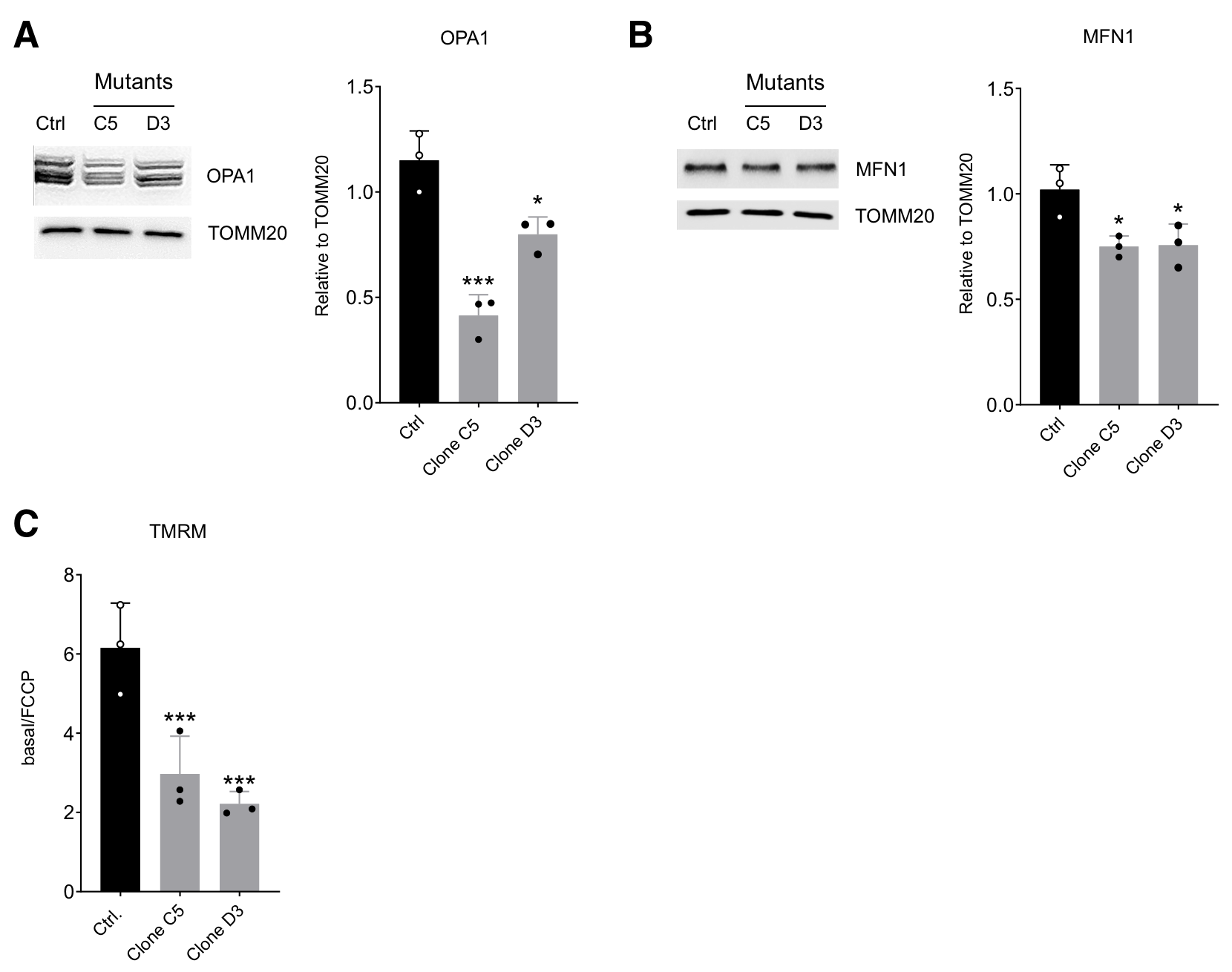

Supplement: Supplementary file 5 — Additional file 5: Figure S2. Mitochondrial membrane potential is altered in Setd5+/− NSCs.Western blot quantification of Opa1and Mfn1protein level in NSCs normalized on Tomm-20. Statistics, Opa1, one-way ANOVA, multiple comparison Dunnet test; Mfn1, one-way ANOVA, multiple comparison Dunnet test.Quantification of mitochondrial membrane potential using TMRM in live imaging. The signal level is calculated by performing a ratio between the basal fluorescence intensity and the fluorescence after FCCP treatment. Statistics, one-way ANOVA, multiple comparison Dunnet test. All data are presented as mean values +/− SEM. [file 13229_2023_550_MOESM5_ESM.tif]

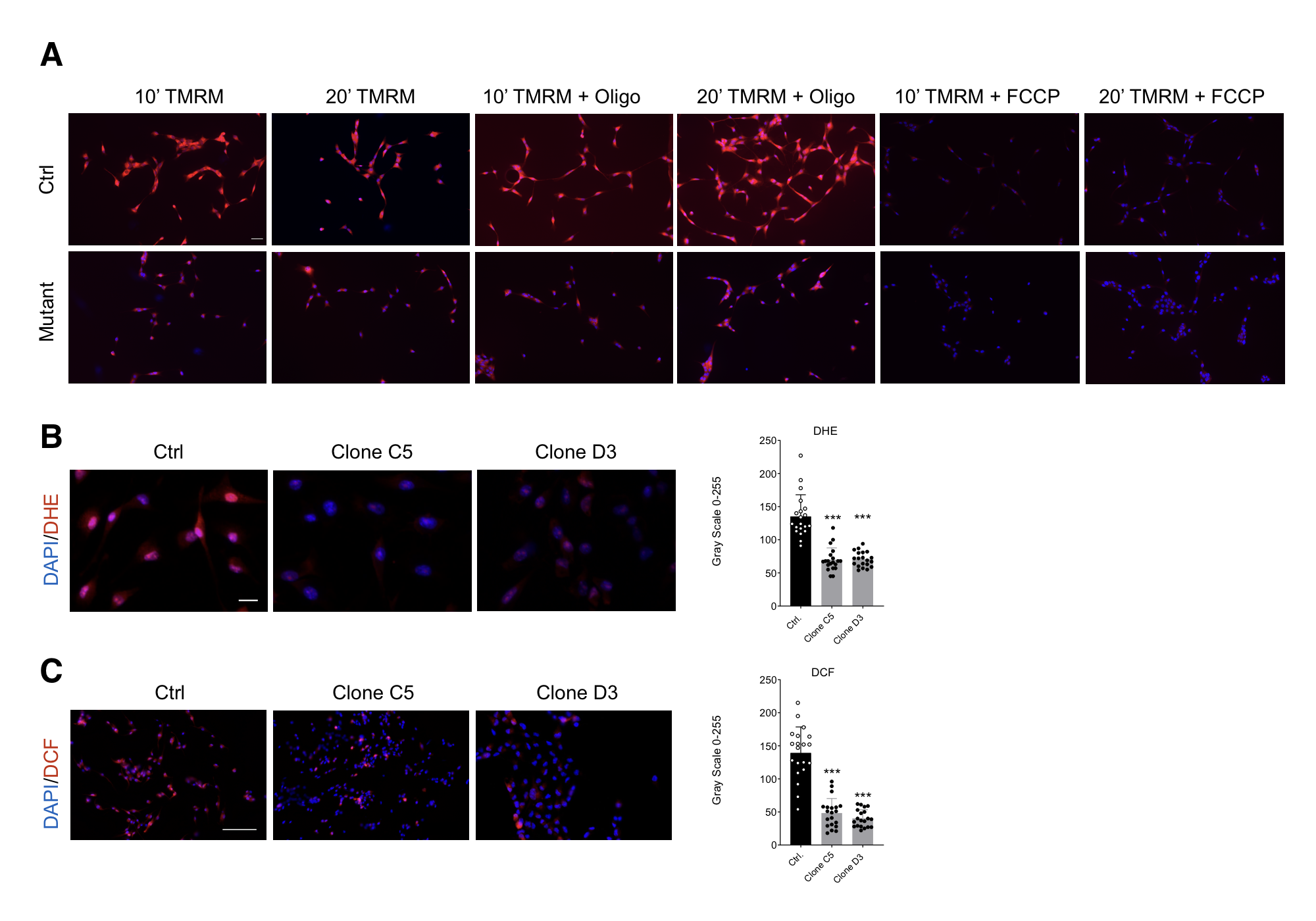

Supplement: Supplementary file 6 — Additional file 6: Figure S3. Mitochondrial function alteration in Setd5+/− NSCs.TMRM live staining on control and Setd5+/− NSCs. From the left, basal condition, oligomycin treatment and last two images FCCP treatment.Reactive oxygen species quantification in control and Setd5+/− NSCs performing live staining of DHEand DCF. Statistics, DHE, one-way ANOVA, multiple comparison Dunnet test; DCF, one-way ANOVA, multiple comparison Dunnet test. All data are presented as mean values +/− SEM. [file 13229_2023_550_MOESM6_ESM.tif]

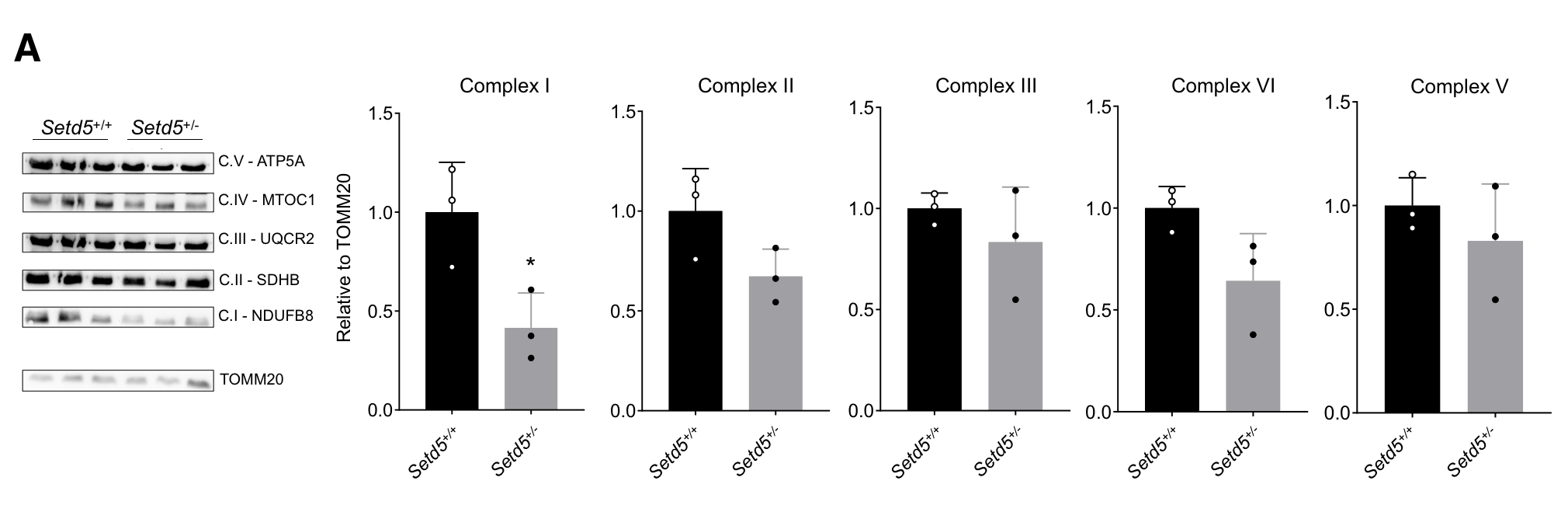

Supplement: Supplementary file 7 — Additional file 7: Figure S4. Electron transport chain alteration in mouse cortex.Western blot analysis on whole protein lysate from Setd5+/+ and Setd5+/− mouse cortex for the indicated mitochondrial protein, normalized on total Tomm-20 level. Statistics, NDUFB8, T-test,; SDHB,; UQCRIII,; MTCO1,; ATP5A,. All data are presented as mean values +/− SEM. [file 13229_2023_550_MOESM7_ESM.tif]

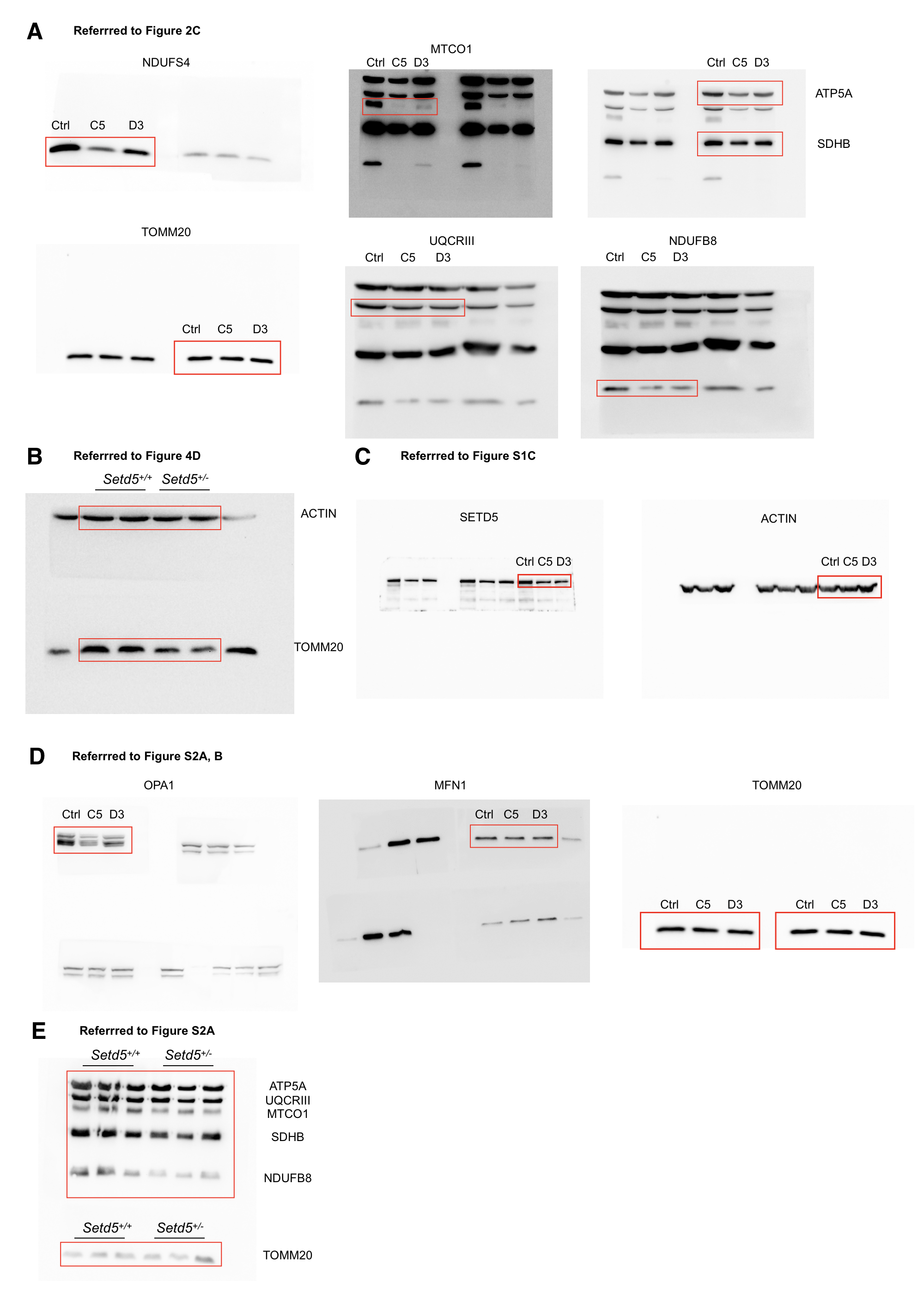

Supplement: Supplementary file 8 — Additional file 8: Figure S5. Uncropped Western blot images.The images refer to Fig. 2C.The images refer to Fig. 4D.The images refer to the figure S1C.The images refer to the figure S2A, B.The images refer to the figure S4A. [file 13229_2023_550_MOESM8_ESM.tif]
